# Exploring *Leishmania* secretory proteins to design B and T cell multi-epitope subunit vaccine using immunoinformatics approach

**DOI:** 10.1038/s41598-017-08842-w

**Published:** 2017-08-15

**Authors:** Nazia Khatoon, Rajan Kumar Pandey, Vijay Kumar Prajapati

**Affiliations:** Department of Biochemistry, School of Life Sciences, Central University of Rajasthan, NH-8, Bandarsindri, Kishangarh, 305817 Ajmer, Rajasthan India

## Abstract

Visceral leishmaniasis (VL) is a fatal form of leishmaniasis which affects 70 countries, worldwide. Increasing drug resistance, HIV co-infection, and poor health system require operative vaccination strategy to control the VL transmission dynamics. Therefore, a holistic approach is needed to generate T and B memory cells to mediate long-term immunity against VL infection. Consequently, immunoinformatics approach was applied to design *Leishmania* secretory protein based multi-epitope subunit vaccine construct consisting of B and T cell epitopes. Further, the physiochemical characterization was performed to check the aliphatic index, theoretical PI, molecular weight, and thermostable nature of vaccine construct. The allergenicity and antigenicity were also predicted to ensure the safety and immunogenic behavior of final vaccine construct. Moreover, homology modeling, followed by molecular docking and molecular dynamics simulation study was also performed to evaluate the binding affinity and stability of receptor (TLR-4) and ligand (vaccine protein) complex. This study warrants the experimental validation to ensure the immunogenicity and safety profile of presented vaccine construct which may be further helpful to control VL infection.

## Introduction

Leishmania parasites are responsible for a family of diseases, known as leishmaniasis with different clinical manifestation ranging from cutaneous lesions to fatal systemic disease^1^. Visceral leishmaniasis (VL) has been classified as one of the most neglected diseases; the estimated disease burden places it second in mortality and fourth in morbidity among the tropical infectious diseases^2, 3^. Currently available antileishmanial treatments are based on chemotherapy but the accessible drugs are toxic, have serious side effects and are related to numerous failures along with increased incidence of drug resistance^4, 5^. There are only a few compounds such as sitamaquine and imidazole that are in pipeline, which still requires pharmaceutical investigations and clinical trials at large level. Foot to mouth situation of people living in the endemic zones is limiting the reach of vector control measures^6, 7^. Development of new vaccines can now take advantage of a much greater knowledge of the complexities of the immune system and of the development of powerful molecular techniques that allow targeted changes to be made in pathogenic organisms and in experimental host^8^. Vaccine development has become much more sophisticated where immunologists working closely with molecular biologists and chemical engineers to design and produce highly purified vaccines that are safe and effective^9^.

The development of a successful vaccine to prevent VL disease condition has been a goal for almost a century, but currently no such vaccine exists^10^. Immunoprophylaxis and vaccination need to develop for the control of this disease^11^. Secretory proteins of pathogens are antigenic in nature and are capable of inducing a significant immunogenic response into the host. Therefore, many vaccine candidates have been derived from secretory proteins of pathogen^12^. B cell epitopes are part of proteins which prime the antigenicity and recognized by the human antibodies. Identification of B and T cell epitopes are useful for the development of a novel vaccine^13^. During the VL infection, T cells become an effector cell and kill an infected cell after the activation of antigen-presenting cells^14^. The peptide fragments of the pathogen and the MHC molecules bind together and appear on the cell surface of infected host cells. A diverse range of peptides is bound to each allelic form of MHC protein^15^. The MHC molecule has the ability to bind with peptides tightly as the pathogens try to mutate the epitope of the MHC molecule. Hence, MHC molecule exhibits high binding affinity with a variety of peptides^16^.

Vaccination, or immunization, work by stimulating the immune system, the natural disease-fighting system of the body^17^. With the discoveries of newer technologies, the conventional empirical approaches to vaccine development have given a way to design rational vaccines^18^. Improved understanding of pathogen variability as well as the diversity of the human immune system, plus technological advances in genetic and molecular biology provide better insights in the host–pathogen interaction and new avenues for vaccine development^19^. In past decade, important advances have been made in the field of the basic immunology. These include the interpretation of the MHC restriction, which concluded in the elucidation of the T cell receptor and the three-dimensional structure of an MHC molecule; the identification of intercellular signaling molecules (cytokines) and the nature of the antigen presentation. However, this application just begins to control the infectious disease. MHC molecules are critical in transplantation, autoimmunity, infections, and tumor immunotherapy. The combined understanding of antigen presentation by MHC molecules allows exploitation to improve the responses of the cellular arm of the immune system towards vaccinations and immunotherapies^20^. Vaccination has been considered as a safest and efficacious way to control the vector-borne parasitic diseases.

In the beginning days, development of immunization against leishmaniasis infection involves the inoculation of live or autoclaved *Leishmania* parasites. But because of safety concern, it is not in practice now for the human use. Several types of the antigens collected from *Leishmania donovani* were used as a vaccine candidate to improve the humoral as well as cell-mediated immune response. These efforts were rejected because of antigenic variability and gene polymorphism in the clinical isolates circulating in the endemic region. Different recombinant proteins which include KMP-11, A2, CPB, and gp63 have proven immunogenic response in the animal model but these were not able to reach up to clinical trials. Leish-111F/MPL-SE is one of the multi-subunit recombinant vaccines which have shown a safe response in South America against cutaneous & mucocutaneous leishmaniasis and cured visceral leishmaniasis patients in India^21^. But still, it is far from the VL clinical trial^22^. Immunoinformatics approach for the identification of novel vaccine is a recently developed method which can predict a thermodynamically stable and multi-epitope subunit vaccine in a very less time. Hence, here we propose to design a multi-epitope subunit model vaccine composed of immunogenic epitopes which may have the ability to activate host humoral and cell-mediated immune system.

## Results

### Collection of *Leishmania donovani* protein sequences for vaccine construction

The amino acid sequence of five secretory proteins of *L. donovani* was retrieved from Gene Bank Database in FASTA format and used for designing a potential multi-epitope vaccine against VL infection. The amino acid sequences of *L. donovani* secretory proteins (LdBPK_190160,hypothetical protein conserved﻿; LdBPK_211410, Surface antigen-like protein; LdBPK_310120, FG-GAP repeat protein; LdBPK_220130, Translocon-associated protein and LdBPK_310860, Secretory lipase) were selected based on their respective D-score which was obtained using SignalP server. Further, 50S ribosomal protein L7/L12 (Locus RL7_MYCTU) P9WHE3 was retrieved from the UniProt database to be used as an adjuvant.

### Cytotoxic T Lymphocytes (CTL) and Helper T Lymphocytes (HTL) epitopes prediction

A total of 39 CTL epitopes (9-mer) were predicted by using NetCTL 1.2 server, among them only 8 epitopes with high ranked binding affinity score were chosen as final CTL epitopes for the input of *L. donovani* secretory protein sequences (Supplementary Table-1). Similarly, the HTL epitopes were identified using IEDB MHC-II prediction module based on the higher binding affinity with MHC- II. The mouse alleles used for the prediction were H2-1Ad, H2-1Ed, and H2-1Ab. HTL epitopes were selected from LdBPK_190160 situated at position 235–249, 221–235 and 761–775; LdBPK_211410 at position 1–15, 451–465 and 127–141; LdBPK_310120 lies at position 743–757, 398–412 and 691–705; LdBPK_220130 found at position 210–224, and 72–86, 260–274; and LdBPK_310860 situated at position 173–187, 247–261 and 135–149 (Supplementary Table-2). Finally, the selected MHC-I and MHC-II binding epitopes were applied to the final selection. Here, MHC-I binding epitopes with high rank were defined as CTL epitopes and similarly, MHC-II binding epitopes with high rank were defined as HTL epitopes.

### Construction of multi-epitope subunit vaccine

**Figure 1:**
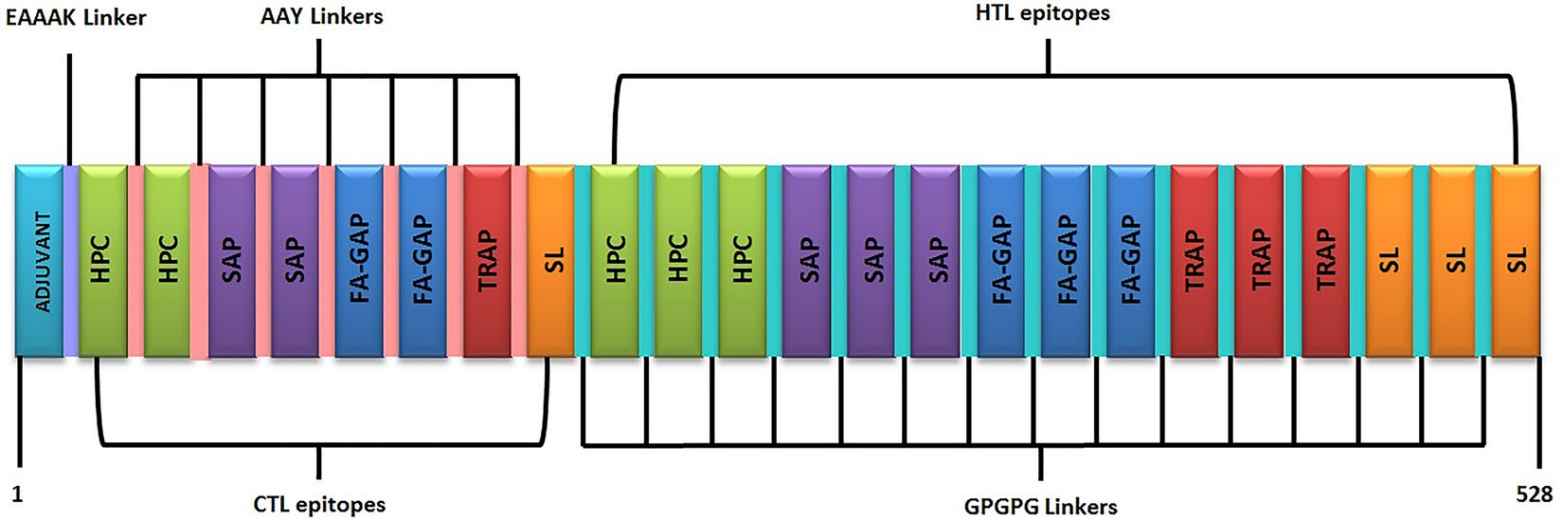
Schematic diagram of final subunit vaccine construct. The multi-epitope vaccine sequence consisting of 528 amino acid residues; out of which, first 130 residues has been represented as adjuvant followed by CTL (131–228 amino acids) and HTL (229–528 amino acids) epitopes. Adjuvant and CTL epitope has been joined by EAAAK linker (Light purple). Whereas, AAY (pink) and GPGPG (cyan) linkers were used to join the CTL and HTL epitopes, respectively. (HPC = Hypothetical protein conserved; SAP = Surface antigen protein; FA-GAP = FA-GAP repeat protein; TRAP = Translocon-associated protein; SL = Secretory lipase).

Based on the score, a total of 8 CTL epitopes and 15 HTL epitopes with high binding affinity were fused together with the help of AAY and GPGPG linkers, respectively, to form the final vaccine construct. Since, expression of TLR4 increases during the *L. donovani* infection in human immune cells, therefore, TLR4 (PDB ID: 4G8A) agonist 50S ribosomal L7/L12 (Locus RL7_MYCTU) with accession no. P9WHE3 was chosen as an adjuvant to potentiate antigen-specific immune responses. The final vaccine constructs composed of 528 amino acid residues as represented in Fig. 1.

### B cell epitopes prediction for *Leishmania donovani* secretory proteins

**Figure 2:**
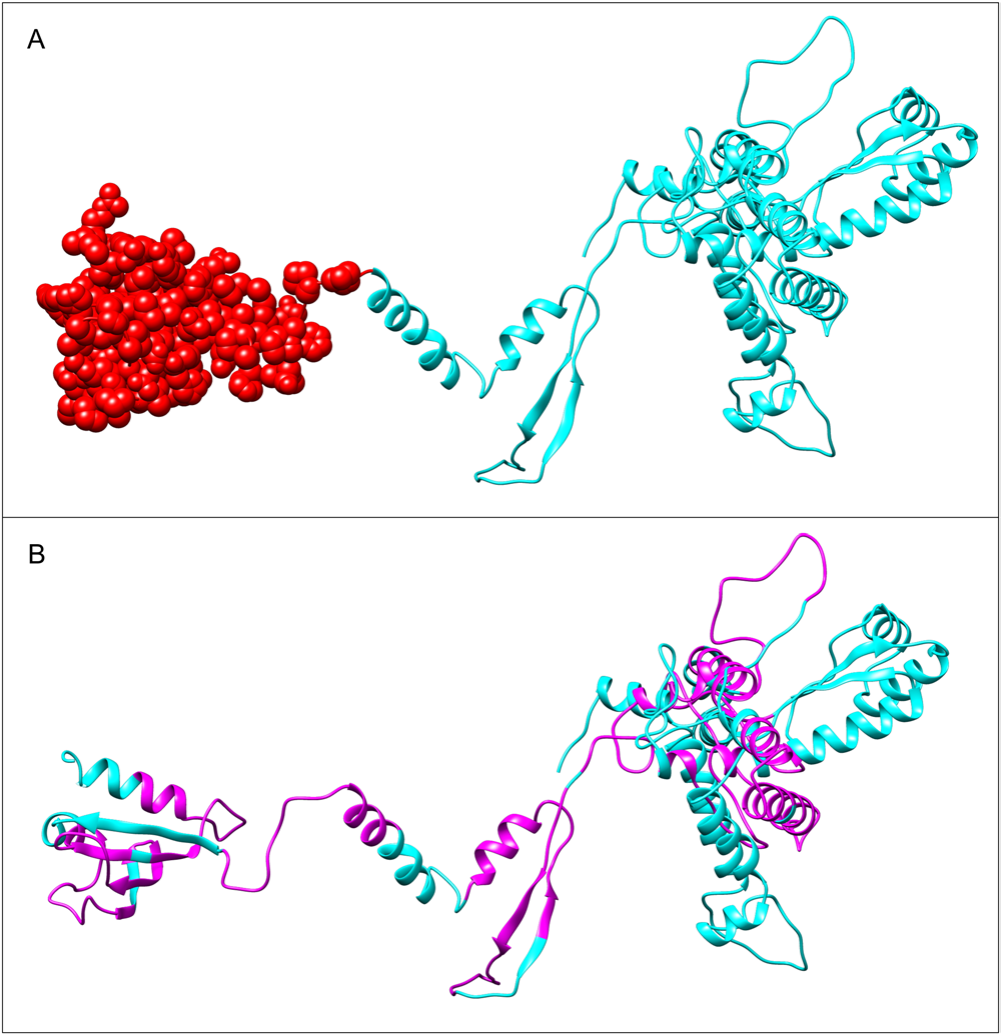
B-cell epitopes prediction for the final subunit vaccine construct. (**A**) The discontinuous B- cell epitopes have been represented as red spheres in the final vaccine model (**B**) linear B- cell epitopes have been shown in magenta color.

BCPREDS server was used to predict the linear B-cell binding epitopes for the final vaccine construct. However, a total of 13 epitopes having 0.99 and above score with 20mer length were chosen for further analysis (Supplementary Table-3). Further, ElliPro suite was utilized to identify the B-cell conformational epitopes. This is based on Thornton’s method and employs both MODELLER program and Jmol viewer for the evaluation and visualization of 3D B cell epitopes of a final vaccine construct sequences. Total 100 residues were predicted as the discontinuous B-cell epitopes that ranging from the residue number 429–528 with a score of 0.817, where the default threshold was 0.5 and the default maximum distance was 6. Moreover, the discontinuous epitope found with start residue Glycine with residue score of 0.557 and end residue was found on Alanine with residue score of 0.970 (Fig. 2A,B).

### Prediction of allergenicity, physiochemical parameter, and antigenicity of the vaccine construct

The vaccine allergenicity was evaluated using AlgPred. The final vaccine construct was found to be non-allergen with the score of −1.06 whereas the default threshold was −0.4. The theoretical isoelectric point value (pI) and molecular weight (MW) of protein were found to be 9.04 and 54.62 kDa, respectively. According, to isoelectric point (pI) the protein is basic in nature. Moreover, the half-life was assessed to be 30 hours in mammalian reticulocytes, *in vitro*; more than 20 hours in yeast and more than 10 hours in *E. coli*, *in vivo*. The computed instability index was found to be 26.3 representing the stable nature of the protein. GRAVY (Grand average of hydropathicity) and the aliphatic index was 83.48 and 0.041 respectively. Further, the antigenicity of final vaccine construct was predicted to be 0.6863% by using the VaxiJen server at 0.4% threshold by selected bacteria model and 0.77% by ANTIGENpro. These results indicated that our final vaccine protein is antigenic in nature.

### Secondary structure prediction

**Figure 3:**
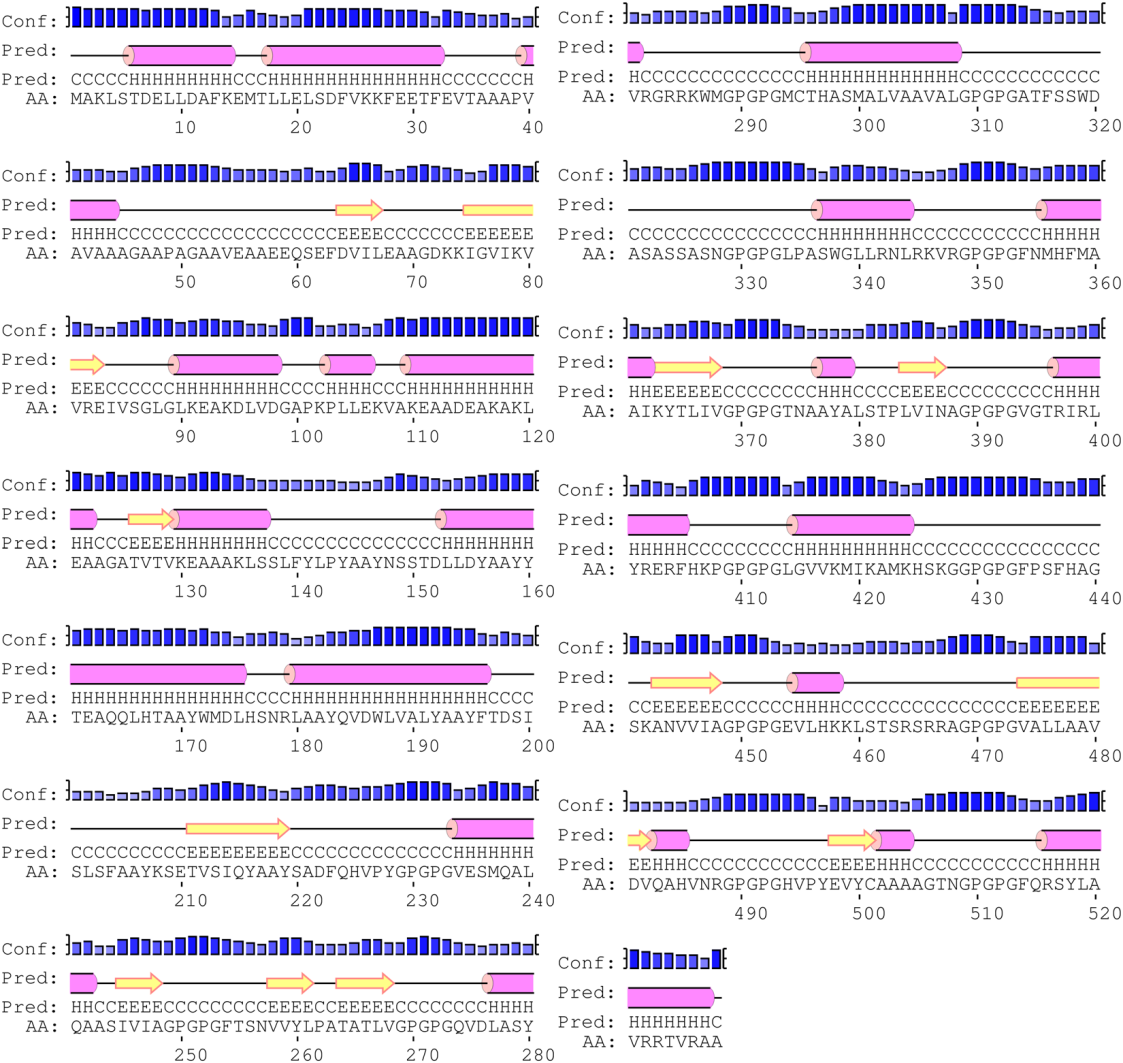
Graphical representation of secondary structure obtained for the final subunit vaccine construct showing alpha-helix (35.8%), beta strands (12.9%) and coils (51.3%).

In view of PSIPRED program secondary structure of final vaccine construct was identified. The predicted structure showing the presence of 35.8% alpha helix, 12.9% beta strand and 51.3% coil structure as shown in Fig. 3.

### Homology modeling and tertiary structure refinement

**Figure 4:**
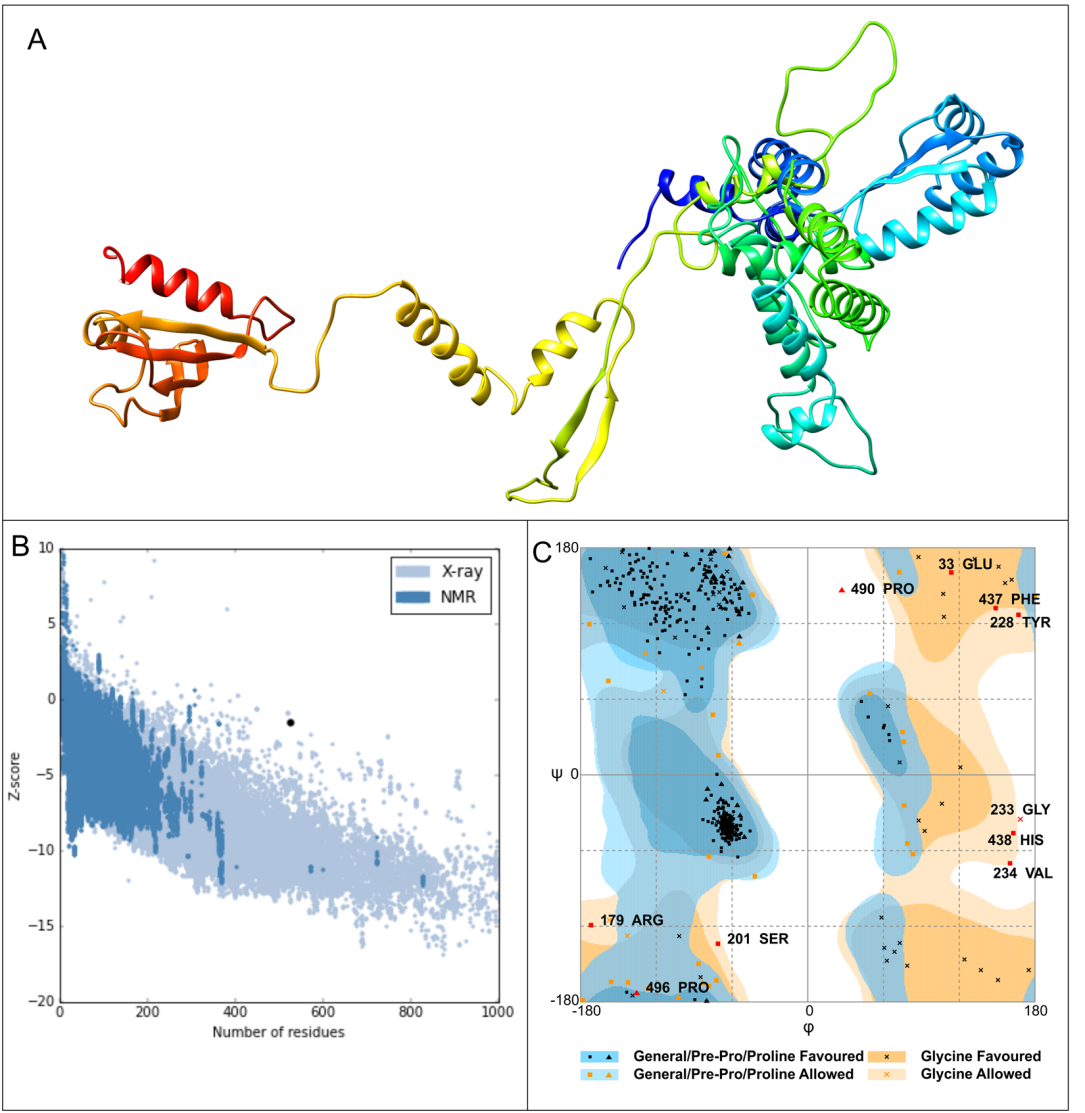
Final subunit vaccine model structure and validation. (**A**) The figure represents final 3D model of multi-epitope vaccine obtained after homology modeling and refinement. (**B**) PROSA validation of 3D structure showing Z-score (−1.52) and (**C**) Ramachandran plot analysis of refined modeled structure showing 92%, 5.9% and 1% residues in favored, allowed and disallowed region respectively.

The 3D model of the final vaccine was generated by using RaptorX program. The output tertiary structure of vaccine protein was partitioned into 3 domains. To model the protein vaccine, multi-template based approach was used for example templates with PDB ID: 1ctfA, 1dd3A, 2ftcE, and 2zjq5 were chosen to model domain1. Similarly, for domain 2 PDB ID: 2mw4A, 3fvyA, 4owwA, 5foiA and 5u71A and domain 3 PDB ID: 1cf3A, 1wooA, 2vycA, 3n75A and 3tfhA were considered. The best template for protein model was (PDB ID: 1dd3A) with its P-value of 3.7e-0.4. The obtained score was 117 with a sequence identity of 51%, rest of the templates was found to be less identical (Fig. 4A). Refinement of the vaccine model was done using GalaxyRefine server. Out of all refined models, model 1 was found to be the best one on the basis of various parameters including GDT-HA (0.9375), RMSD (0.355), MolProbity (2.099), Clash score (14.0), Poor rotamers (0.7) and Ramachandran plot (92.0). This model was taken as the final vaccine model for the further analysis.

### Tertiary structure validation

To validate the 3D models of vaccine protein, the distributions of Ramachandran maps were depicted after homology modeling processes. The Ramachandran plot analysis of a modeled protein revealed that 92% of residues are located in most favored regions, 5.9% in allowed regions and only 1% in disallowed region. The quality and potential errors in a crude 3D model were verified by ERRAT and ProSA-web. The overall quality factor of a modeled protein was 70.3% using ERRAT. While ProSA-web has shown the Z-score of −1.52 for the input vaccine protein model which is lying outside the scores range that commonly found in the case of native proteins of comparable size (Fig. 4B,C).

### Molecular docking of subunit vaccine with immune receptor (TLR-4)

**Figure 5:**
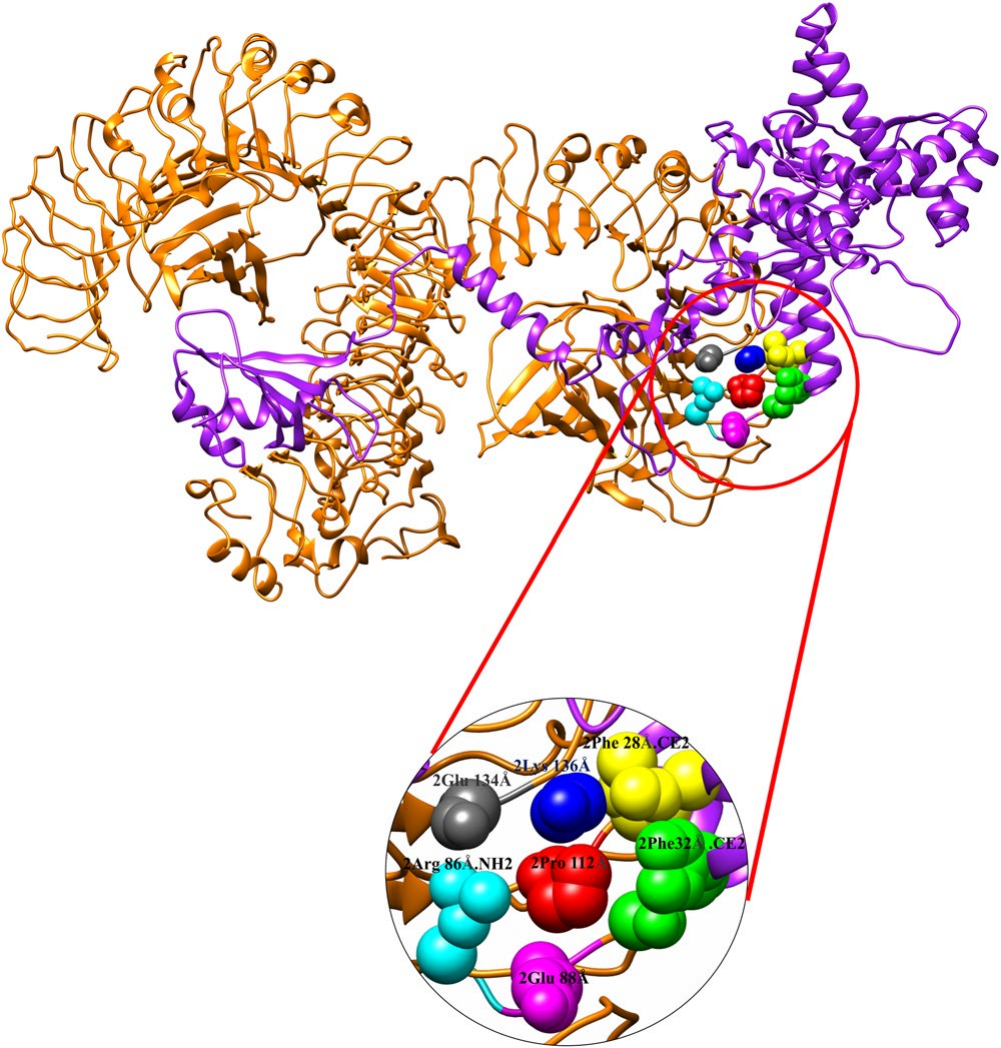
Binding site prediction by using CASTp server. Total two binding sites has been obtained where Grey, blue and yellow color residues showing the first binding pocket; green, magenta, cyan and red colored amino acid residues representing the second binding site.

**Figure 6:**
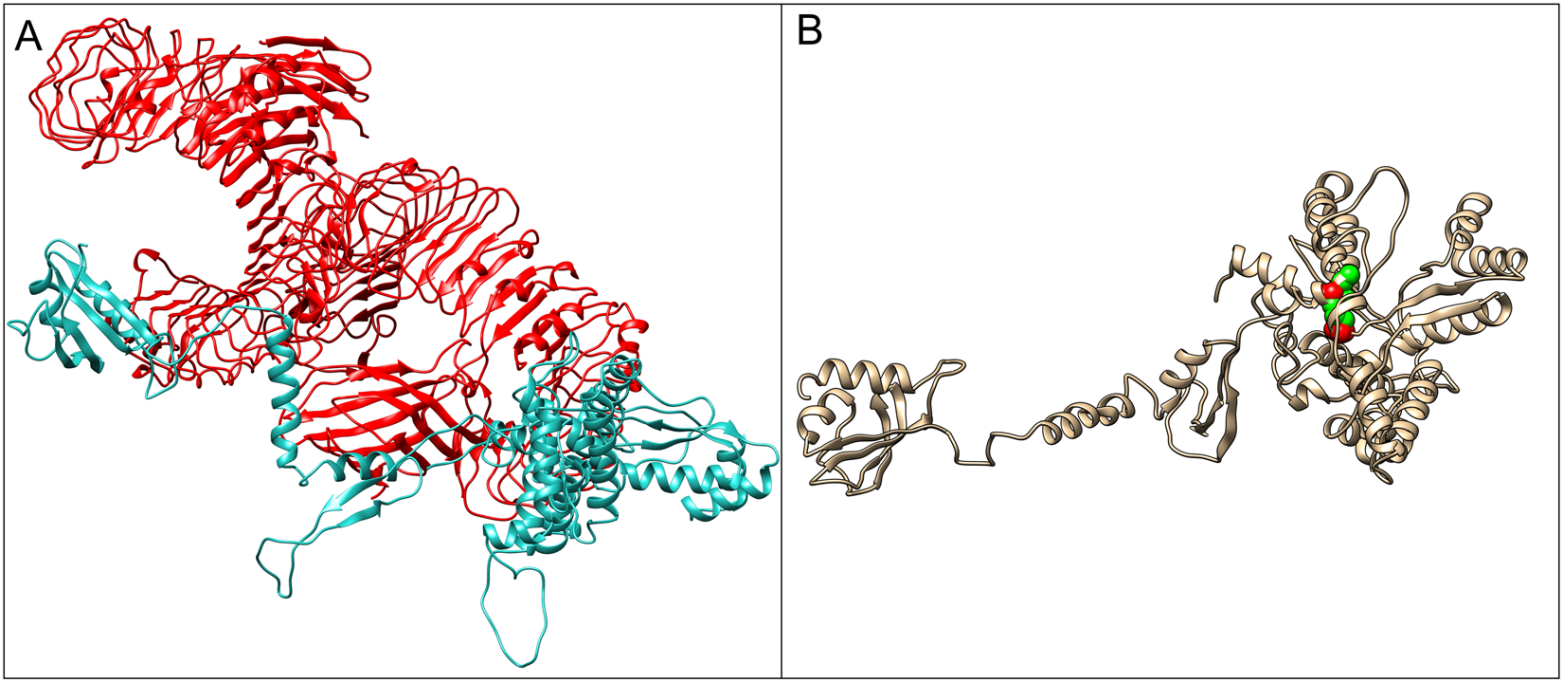
Docked complex of TLR-4 (PDB ID: 4G8A) with subunit vaccine construct. (**A**) Receptor (TLR-4) has been shown in red color whereas cyan color represents the multi-epitope vaccine as a ligand in the docked complex obtained from PatchDock server. (**B**) Representing disulfide bond engineered in the most flexible region with high B-factor.

To determine the protein binding site and hydrophobic interaction on the protein surface, CASTp server was used. Binding pocket was identified at the residues number 1–298 that may act as a potential binding site in TLR4. The pocket molecular surface area was 325.1 Å^2^, its molecular surface volume was 581.3 Å^3^, mouth molecular area was about 197.8 Å^2^ and molecular circumference sum was 80 Å. The two opening pockets were found with residues Arg 86 , Cys87 , Glu88, Ile89 , Glu90 , Gly110, Asn11 , Pro112 , Glu134 , Thr135 , Lys136 , Val40, Pro39 , Ala37 , Ala36, Thr35 , Thr31 , Phe32 , Ala38  and Phe28 (Fig. 5). Protein vaccine-mediated targeted docking against TLR4 was performed by PatchDock server at the predicted binding pocket (Fig. 6A). This generated top 10 models, which were scored according to geometry and electrostatic complementarity of the protein surface. For refinement and re-scoring of molecular docking solutions, FireDock (Fast Interaction Refinement in Molecular Docking) server was used. The refined candidates were ranked by their respective binding energy. Among, the top 10 models output of FireDock, final model was chosen for molecular docking based on the binding score. This score includes atomic contact energy (1.05), Van Der Waals interactions (−15.09), partial electrostatics (6.12) and additional estimations of the binding free energy (−11.97).

### Disulfide engineering of final vaccine constructs

Disulfide by Design^23^ (DbD) resulted in 62 pairs of amino acid sites for probable disulfide bridges. But, only two native disulfide bridges were identified based on their high B-factor and their presence in the spanning region of high mobility. By substituting residues Val54-Ser337; Glu59-Trp338 with cysteine in the final vaccine construct, thermal stability was improved. Furthermore, these mutations were created by using PyMOL^24^ visualization program and the mutant form was generated (Fig. 6B).

### Molecular dynamics simulation of immune receptor-vaccine complex

**Figure 7:**
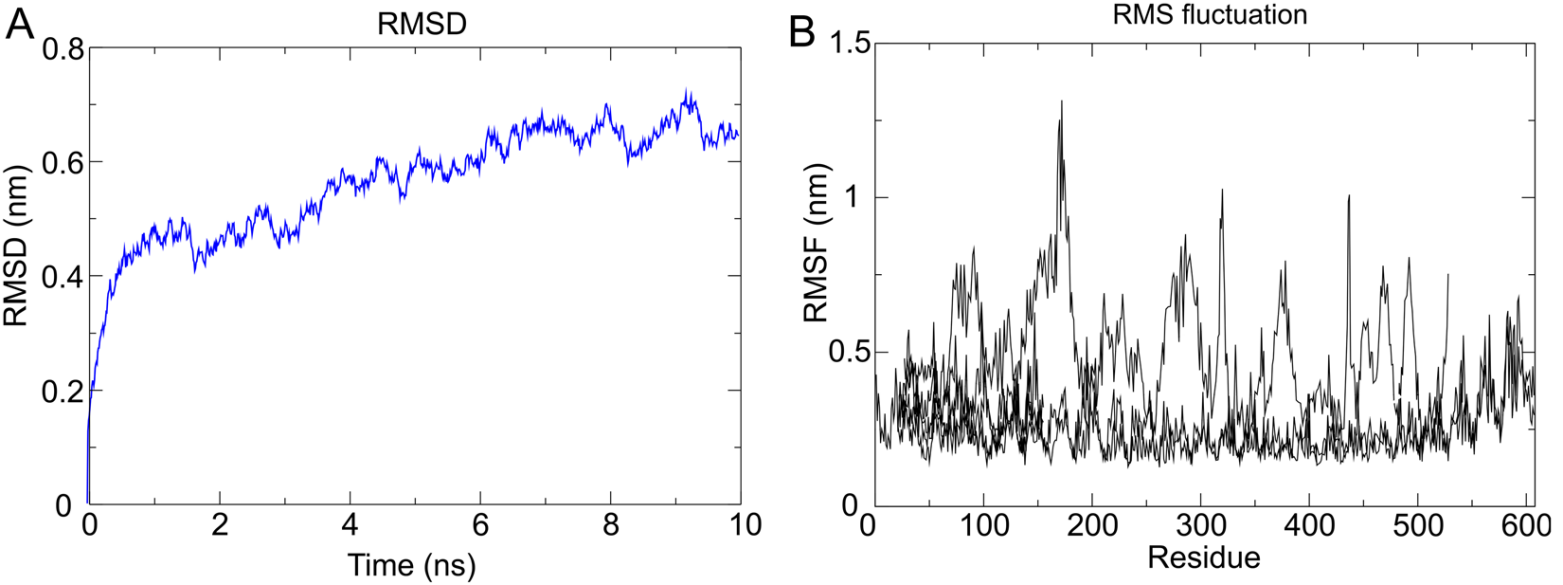
Molecular dynamics simulation study of protein-ligand complex representing. (**A**) Root Mean Square Deviation of the docked complex backbone for the time duration of 10 ns. (**B**) Root Mean Square Fluctuation representation of the docked complex side chains for same time duration.

To study, the physical movements of atoms and molecules of final vaccine construct molecular dynamics simulation was performed as described elsewhere^25–27^. The energy components, temperature, pressure, density and volume of the simulation systems were evaluated and their stabilities were confirmed. The protein vaccine energy was minimized with the steepest descents converged to f_max_ 1000 in 1925 steps having the potential energy of 14976.4 KJ/mol when it runs for 2 nano-seconds (ns) (Supplementary Figure 1). Further, to stabilize the pressure and temperature of the system, equilibration of temperature and pressure was performed where n was equal to a number of particles while pressure and temperature were kept constant (Supplementary Figure 2). In order to evaluate the stability of final vaccine construct, the root square deviation (RMSD) of the protein backbone and the root mean square fluctuation (RMSF) of all side chain residues were analyzed for the time period of 10 ns to identify whether the structure shows stability or not. The RMSD of the TLR4 receptor and multi-epitope vaccine protein complex was 6 Å (Fig. 7A). The fluctuation of the side chain residue ranging from 5 Å to 10 Å with a little variation (Fig. 7B). Total two hydrogen bonds were formed between the protein-ligand complexes where the first bond was formed between Lys263 and Asp 101 with a distance of 2.047 Å and another was formed between Tyr102 and Phe262 at a distance of 1.789 Å.

### Codon optimization of final vaccine constructs

**Figure 8:**
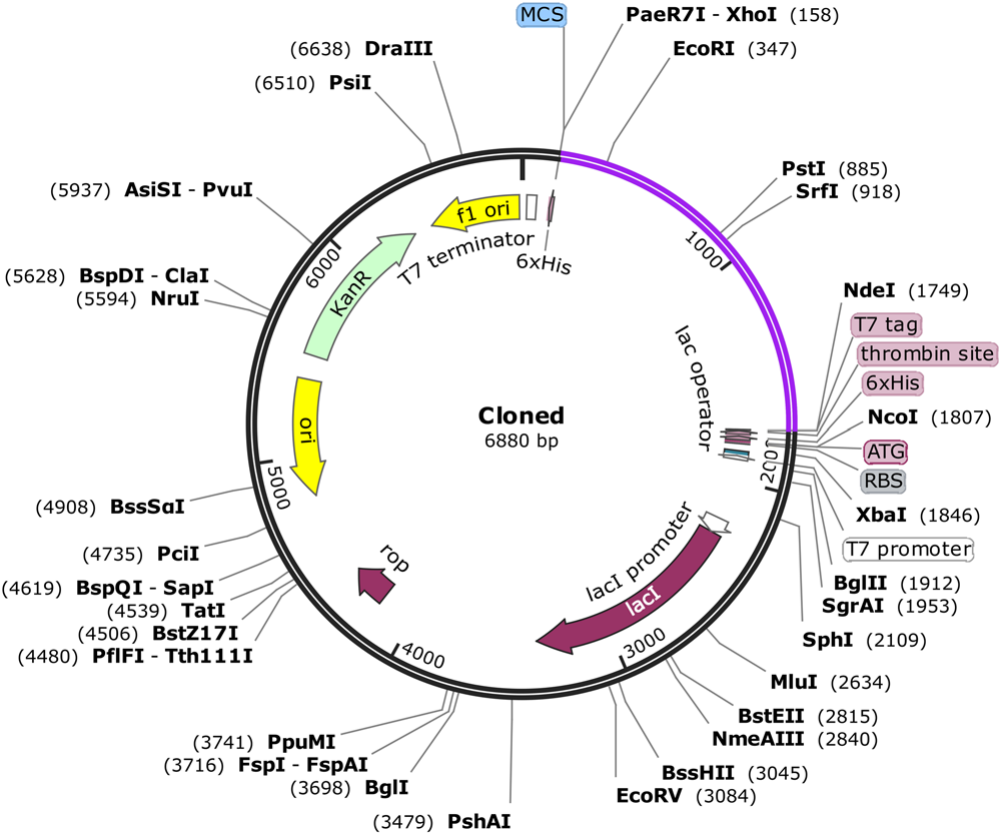
*In silico* restriction cloning of final vaccine construct into pET28a(+) expression vector where purple part representing the vaccine insert and black circle showing the vector.

To optimize the codon usage of vaccine construct in *E. coli* (strain K12), Java Codon Adaptation Tool was used and the maximal protein expression was assured. The length of optimized codon sequence was 1584 nucleotides. Codon Adaptation Index (CAI) of optimized nucleotide sequence was 0.95 and the average GC content of our sequence was 55% showing good expression possibility of vaccine protein in *E. coli* host. The optimal percentage range of GC content should lie in between 30% to 70%. Finally, restriction clone was formed by inserting the adapted codon sequences in pET28a(+) vector (Fig. 8)

## Discussion

Vaccination is one of the most effective means to efficiently, rapidly and affordably improve the public health and the best way to control infectious disease in the society. Current research is focused on subunit vaccines as compared to whole organism vaccines because subunit vaccines contain specific immunogenic components of the pathogens responsible for the infection rather than the whole pathogenic agent. In the recent decades, the genomic and proteomic information of *Leishmania* and other pathogenic microbes is available in bulk. The information from the data is helpful to identify potential antigenic targets using computational approaches.

The secretory proteins of *Leishmania* have diverse functions during visceral leishmaniasis infection^28^. First, they play a role in the formation of the infection in conjunction with important elements present in the saliva of the sand fly vector. In a second phase, they contribute to maintain the infection by interfering with the macrophage microbicidal functions, cytokine production, antigen presentation, and effector cells activation. The identification of *L. donovani* secretory proteins antigenic epitopes and understanding their interactions with the host MHC alleles and immune cell have huge immunological values, which could be used for the development of vaccines against *L. donovani* ^29^.

In our study, five *L. donovani* secretory proteins were identified and scanned for the presence of a signal peptide using TARGET p1.1 program. All proteins were found to be secretory protein on the basis of D-score which introduced in signal peptide and is a simple average of the S-mean and Y-max score^30^. *L. donovani* secretory proteins were further used to carry out a detailed analysis with the aim of evaluating the immunogenic potential of these proteins. In order to identify the *Leishmania* epitopes showing shared sequence with the host proteome, *L. donovani* secretory proteins were subjected to BLASTp search against host proteomes of humans. The secretory proteins from *L. donovani* did not show any homology with any of the proteins from the human host. This supports the argument that the *L. donovani* secretory proteins are perfect antigenic targets for trial against Leishmaniasis. Further, B and T cell epitopes were collected from the identified secretory proteins. T cells identify the peptide epitopes presented by the MHC molecules, which are the surface proteins of antigen presenting cells recognized by the T cell receptors (TCR)^31^. These antigen-presenting molecules (MHC) are either class I or II. MHC class I molecules present on nearly all nucleated cells that specifically represents the endogenous proteins or antigen that processed through the cytosolic pathway and represents towards cytotoxic T lymphocytes (CTLs). MHC class II molecules present exogenous antigens, generally surface proteins of the pathogens which are processed through endocytic pathways and presented to helper T lymphocytes or CD4+ T cells. The immunoinformatics analysis shows that our final vaccine protein contains a large number of high affinities MHC Class I and MHC Class II and B-cell linear epitope based on physiochemical property and structural features. Antigenicity and Allergen property of final protein vaccine make it a potent vaccine. The molecular weight of vaccine protein was 54.62 kDa, showing an average molecular weight to construct a multi-subunit vaccine, theoretical pI was 9.04 which allow to vaccine protein to be basic in nature, aliphatic index shows protein is occupied by aliphatic side chains, instability index classifies that vaccine protein is stable, Grand average of hydropathicity (GRAVY) predicted 0.041 shows that vaccine protein is hydrophobic in nature. All these parameters depicting that it may be thermostable protein. PSIPRED V3.3 is a popular and highly accurate method which has been used in this research for evaluation of vaccine secondary structure. Further, the 3-dimensional structure obtained by homology modeling contains sufficient information about the spatial arrangement of important residues in the proteins and great assistance in the study of protein function, dynamics, interaction with ligand and other proteins. Structure validation tools were used to recognize errors in modeled structure of final vaccine construct. Ramachandran plot shows the most of the residues clustered tightly in the most favored region with very few residues in outliers which depicted that the overall model quality is satisfactory. Furthermore, to know the immune response of TLR-4 agonists against vaccine construct, we performed docking analysis. For overall conformational stability of vaccine protein-TLR4 docked complex, energy minimization was performed to minimize the potential energy of the complete system. Energy minimization repairs the unnecessary geometry of structure by replacing some of the protein atoms and hence forms a more stable structure with suitable stereochemistry. The predicted RMSD of the vaccine-TLR-4 complex was found to be 6 Å, which shows the stability of the complex.

To attain high-level expression of recombinant vaccine protein in *E. coli* (strain K12), codon optimization was carried out to improve transcriptional and translational efficiency^32^. This was accomplished by analyzing codon adaptation index (CAI) and total GC content of DNA sequence. The solubility of the overexpressed recombinant protein in the *E. coli* host is one of the essential requirements of many biochemical and functional investigations. Our protein vaccine shows an admissible percentage of solubility in an overexpressed state. Enhancing the strength of proteins is an imperative target in numerous biomedical and mechanical applications. In this study, we brought novel disulfide bonds into multi-subunit vaccine construct to enhance thermostability of the protein, adjust practical characteristics and to help in the investigation of protein elements. Previously designed and tested vaccines in experimental animal model have shown good immunogenic response but because of complex nature of human immunopathology, none of them have shown same response when tested in human. Therefore, in this study, we have integrated novel immunoinformatics tools to design a potential, safe and immunogenic subunit vaccine which may have the ability to control the visceral leishmaniasis infection.

## Conclusion

Visceral leishmaniasis infection has emerged as a severe problem at a worldwide level and reasons for a large number of deaths in tropical and sub-tropical regions across the globe. Despite this, there is no permanent cure and prevention for the VL infection. In this work, we have attempted to create a multi-epitope based subunit vaccine through immunoinformatics approaches. This study starts with the retrieval of five *L. donovani* secretory proteins followed by the prediction of immunogenic B-cell and T-cell epitope to generate the humoral and cell-mediated immunity, respectively. Predicted epitopes were merged together by using suitable linkers and adjuvant to enhance the immunogenicity and effective separation of epitopes within the human body. Allergenicity and antigenicity were also confirmed followed by the physiochemical properties evaluation. Molecular docking and dynamics were also performed to check the binding affinity and stability of TLR-4 and vaccine complex. At last, disulfide engineering and *in silico* cloning was performed to ensure the stability and effective expression of vaccine construct. This study utilizes a series of immunoinformatics approaches to generate a multi-epitope based subunit vaccine construct. Furthermore, the proposed vaccine needs to be experimentally validated to ensure the control of VL infection by generating an effective immunological memory.

## Methodology

### Collection of *Leishmania donovani* secretory protein for vaccine construction

The complete amino acid sequences of five secretory proteins of *L. donovani* (LdBPK_190160, LdBPK_211410, LdBPK_310120), LdBPK_220130 and LdBPK_310860) were retrieved from Gene Bank Database (http://www.genedb.org/Homepage). These proteins were selected on the basis of D-score which was predicted using Signal P server, which is a simple average of the S-mean and Y-max score. The score shows superior discrimination performance of secretory and non-secretory proteins to that of the S-mean score which was used in SignalP 4.1 server. For non-secretory proteins, all the scores represented in the signal P3-NN output should ideally be very low.

### Cytotoxic T lymphocytes (CTL) epitope prediction

CTL epitopes for all secretory proteins of *L. donovan*i were predicted using NetCTL 1.2 server (http://www.cbs.dtu.dk/services/NetCTL/). The prediction was based on three main components namely prediction of MHC-I binding peptides, proteasomal C-terminal cleavage, and TAP (Transporter Associated with Antigen Processing) transport efficiency. MHC-I binding and proteasomal C-terminal cleavage were achieved by the utilization of artificial neural networks whereas prediction of TAP transporter efficiency was done by the weight matrix. The threshold value for epitope identification was set as 0.75 during prediction of CTL epitopes.

### Helper T-cell (HTL) epitope prediction

HTL epitopes of 15-mer length for mouse alleles (IAb, IAd, IAs, IEb, IEd and IEs) were predicted for secretory proteins of *L. donovani* using Immune Epitope Database (IEDB) (http://www.iedb.org/). The IEDB server is based on the affinity for their receptor that can be inferred from the IC_50_ value assigned to each epitope. Peptides with higher affinity should have IC_50_ values <50 nM. The IC_50_ value <500 nM indicates an intermediate affinity while <5000 nM can be directly related to the low affinity of epitopes. The value of percentile rank can be inversely related to the affinity for each epitope.

### B cell epitope prediction

B cell epitopes are antigenic determinants that are recognized and bound by receptors present on the surface of B lymphocytes. B-cell epitope prediction followed by its characterization leads to an essential part of antibody plan. BCPred (B cell epitope prediction) server (http://ailab.ist.psu.edu/bcpred/) is a novel method for predicting linear B-cell epitopes by using kernel methods. These are a class of algorithms for pattern analysis, whose best-known member is the SVM (support vector machine). The predictive performance of BCPred (AUC = 0.758) and its output performance is based on SVM as well as the implementation of AAP (Amino acid pair antigenicity) (AUC = 0.7)^33^. This method predicts linear B-cell epitopes using AAP. Further, for conformational B-cell epitopes prediction, ElliPro web tool (http://tools.iedb.org/ellipro/) was used. ElliPro is a structure-based approach for the prediction of antibody epitopes. It implements the result on the basis of Thornton’s method and residue clustering algorithm, the Modeller program and Jmol viewer, used for the prediction and visualization of antibody epitopes in a given structure. ElliPro associates each predicted epitope with a score, defined as a PI (protrusion index) value. According to this, it defines the 3D shape by a number of ellipsoids^34^. For each residue, a PI value is well defined based on the residue’s center of mass which is situated outside the largest possible ellipsoid. Residues with larger scores are related to greater solvent accessibility. Discontinuous epitopes based on PI value as well as clustered algorithms i.e. based on distance R lies in between residues center of mass. The residues with larger distance are linked with the larger discontinuous epitopes being predicted.

### Construction of multi-epitope vaccine sequence

From the above immunoinformatics prediction, a vaccine sequence was constructed using high scoring CTL and HTL epitopes. These epitopes were linked together by using AAY and GPGPG linkers, respectively. Further, 50S ribosomal protein L7/L12 (Locus RL7_MYCTU) was taken as adjuvant (Accession no. P9WHE3) to improve the immunogenicity of the vaccine added at N-terminal and its sequences were retrieved from the National Centre for Biotechnology Information (https://www.ncbi.nlm.nih.gov/).

### Allergenicity Prediction of vaccine

In order to predict multi-epitope vaccine allergenicity with high accuracy, a combination of the different algorithm (SVMc+ IgEepitope+ ARPs BLAST+ MAST) was exploited using AlgPred web tool (http://www.imtech.res.in/raghava/algpred/). The precision obtained for this hybrid algorithm is nearly 85% at the threshold of 0.4. This server performs the prediction based on six different approaches. These approaches can be used to predict allergenic proteins with a high accuracy.

### Antigenicity Prediction of vaccine

ANTIGENpro is a sequence-based alignment-free and pathogen-independent predictor, using protein antigenicity microarray data for the prediction of protein antigenicity. It is accessible online at (http://scratch.proteomics.ics.uci.edu/) and was used to generate an antigenicity index. The server predicts antigenicity on the basis of 2 approaches i.e. multiple representations of the primary sequence and five machine learning algorithms which predict antigenicity based on the obtained results by protein microarray data analysis.

### Physiochemical Properties and Domain Identification

Various physiochemical parameters which involve amino acid composition, theoretical pI, instability index, *in vitro* and *in vivo* half-life, aliphatic index, molecular weight and grand average of hydropathicity (GRAVY) were assessed by using online web server namely ProtParam at (http://web.expasy.org/protparam/)^35^.

### Secondary Structure Prediction

PSIPRED is a web-based freely accessible online server (http://bioinf.cs.ucl.ac.uk/psipred/) that predicts the protein secondary structure for the input of primary amino acid sequences in a precise manner. Position-specific iterated BLAST (Psi-Blast) was performed to identify and select the related sequences showing significant homology to the vaccine protein. Utilizing an exceptionally stringent cross-approval strategy to assess the technique’s execution, PSIPRED 3.2 accomplishes a normal Q3 score of 81.6%. The selected sequences were utilized to construct a position-specific scoring matrix. This built matrix was further processed by two feed forward neural network constructed and trained for the prediction of secondary structure of the vaccine protein as input sequences.

### Tertiary structure prediction

Homology modeling of the final multi-epitope vaccine was carried out by using RaptorX server (http://raptorx.uchicago.edu/). RaptorX predicts secondary and tertiary structures, contacts, solvent accessibility, disordered regions and binding sites. RaptorX also assigns some confidence scores to indicate the quality of a predicted 3D model: P-value for the relative global quality, GDT (global distance test) and uGDT (un-normalized GDT) for the absolute global quality, and modeling error at each residue. CHIMERA 1.11 was used for visualization of modeled 3D structure proteins.

### Refinement of the Tertiary Structure

The refinement process was done by using Galaxy Refine server at (http://galaxy.seoklab.org/) in order to refine the multi-epitope vaccine construct. This server depends on CASP10 based refinement technique to reconstruct the protein side chain followed by repacking and molecular dynamics simulation to relax the structure. As per the CASP10 evaluation, GalaxyRefine is one of the best performing algorithms to enhance the local structural quality. But, this can have the capability to improve both the global as well as local structural quality by refining the models that are generated by using the state-of-the-art protein model prediction servers

### Tertiary structure validation

Model validation is one of the most important steps in the model building sequence. Here, we utilized ProSA-web (https://prosa.services.came.sbg.ac.at/prosa.php) that provides an easy-to-use interface to the program ProSA which is frequently employed in protein tertiary structure validation. ProSA calculates an overall quality score for a specific input structure. If this score is outside a range characteristic for native proteins the structure probably contains errors. A plot of local quality score point to the problematic part of the model which is also highlighted in a 3D molecule viewer to facilitate their detection. ERRAT server (http://services.mbi.ucla.edu/ERRAT/)^36^ was used to analyze the statistics of non-bonded interactions. Ramachandran plot was analyzed by using RAMPAGE server (http://mordred.bioc.cam.ac.uk/~rapper/rampage.php). This server uses PROCHECK principle to validate a protein structure by using Ramachandran plot and separates plots for Glycine and Proline residues.

### Molecular docking of vaccine with receptor (TLR-4)

The binding pocket or cavities among the TLR-4 receptor was predicted by using CASTp server (http://sts.bioe.uic.edu/castp/). Alpha shape theory based pocket algorithm make dependency of CASTp server for the pocket prediction. CASTp has an excellence for providing identification and measurements of surface accessible binding pockets along with the information of inner inaccessible cavities for protein molecule. The calculation uses a solvent probe of radius 1.4 Å. Currently, the server does not support any other probe radius or Vander-Waals molecules. Molecular docking of the final vaccine construct with TLR4 (PDB ID: 4G8A) receptor was performed using PatchDock (http://bioinfo3d.cs.tau.ac.il/PatchDock/) to show the development of immune response. PatchDock is a calculation for atomic docking. The calculation is in view of three noteworthy stages i.e. atomic shape portrayal, surface fix coordinating and separating and scoring. PatchDock divides the surface of both input molecules into patches in accordance with the shape of the surface. These patches then further correspond to specific patterns that can visually distinguish between puzzle pieces. After identification of these patches, their superimposition is achieved by using shape matching algorithms. For more, refinement and re-scoring of rigid body molecular docking solutions FireDock (Fast Interaction Refinement in Molecular Docking) server was used. It gives best 10 solutions for final refinement. The refined models were based on the binding score. This score includes Atomic contact energy, Van Der Waals interaction, partial electrostatics and estimations of the binding energy.

### Disulfide engineering of final vaccine construct

The *in silico* plan of disulfide engineering is used to provide strength to 3D vaccine construct was performed by utilizing Disulfide by Design 2 server. It helps in increasing the protein stability along with the examination of protein interactions and dynamics. The increasing stability of protein after disulfide engineering is due to reduced conformational entropy of unfolded protein state. The spanning region of high mobility was selected based on the obtained B-factor followed by the creation of four stabilizing mutations to make a disulfide bridge. The B-factor is a measure of dynamic mobility of atom in a given protein.

### Molecular dynamics simulation of receptor-ligand complex

Molecular dynamics simulation is an important step in order to determine the stability of protein-ligand structure as described elsewhere^37–42^. Here, molecular dynamics simulation of the multi-epitope vaccine was carried out using Groningen Machine for Chemical Simulations (GROMACS) 5.0 software packages^43^. Force field applied for the further analysis was GROMOS 96 43a1, this force field used to determine the intermolecular interactions during the MD simulation process. Energy minimization was performed to remove very high energy configurations (steric clashes or bonded terms far from equilibrium). Some large protein structure may cause bad contacts, unphysical changes in potential energy in subsequent dynamics simulations so a brief minimization can remove such potential problems. Afterward, systems go through with NVT ensemble MD simulation at 300 K temperature for the duration of 100 ps. Similarly, NPT equilibrium runs at 300 K and 1 bar pressure. MD simulation was performed on the chosen conformation of the receptor-vaccine complex in the docking process for 10 ns. Including the correction, maps improve sampling of near-native state conformations in our systems, and to some extent, it is even able to refine distorted protein conformations. RMSD (root mean square deviation) and RMSF (root mean square fluctuation) were carried out to check the standard deviation and fluctuation of the protein backbone.

### *In silico* cloning optimization of vaccine protein

To express the multi-epitope vaccine construct in a proper expression vector, reverse translation and codon optimization were performed using Java Codon Adaptation Tool (JCat). Codon optimization was performed to express the final vaccine construct in *E. coli* (strain K12) host. Because the codon usage of *E. coli* differs from the native host *L. donovani* from where the sequence of final vaccine construct arises. Three additional options were selected to avoid the rho-independent transcription termination, prokaryote ribosome binding site and restriction enzymes cleavage sites. JCat output includes codon adaptation index (CAI) and percentage GC content can be used to ensure the high-level protein expression. Moreover, to clone the adapted gene sequence of final vaccine construct in *E. coli* pET-28a(+) vector, XhoI and NdeI restriction sites were introduced to the N and C-terminal of the sequence, respectively. At last, the optimized sequence (with restriction sites) was inserted into the pET-28a(+) vector using SnapGene tool to ensure the vaccine expression.

## Electronic supplementary material

Supplementary Info
