# Supplementary material for: Exploring Leishmania secretory proteins to design B and T cell multi-epitope subunit vaccine using immunoinformatics approach
Source: Sci Rep. 2017 Aug 15;7:8285. doi: 10.1038/s41598-017-08842-w (PMC5557753; doi:10.1038/s41598-017-08842-w)
Supplement: Supplementary file 1 — Supplementary Info [file 41598_2017_8842_MOESM1_ESM.pdf]

Exploring *Leishmania* secretory proteins to design B and T cell multi-epitope subunit vaccine using immunoinformatics approach

Nazia Khatoon[#], Rajan Kumar Pandey[#], Vijay Kumar Prajapati*

Department of Biochemistry, School of Life Sciences, Central University of Rajasthan, NH-8, Bandarsindri, Kishangarh, 305817, Ajmer, Rajasthan, India

[#]Contributed equally to this work.

***Corresponding author address**

Dr. Vijay Kumar Prajapati
Department of Biochemistry,
School of Life Sciences,
Central University of Rajasthan,
NH-8, Bandarsindri, Kishangarh,
305817, Ajmer, Rajasthan, India
Email- vkprajapati@curaj.ac.in

Supplementary table 1: Predicted CTL epitopes for *Leishmania donovani* secretory proteins

S. No.	Sequence id	Epitopes	Score
**1**	**LdBPK_190160**	TTGIARGAY	2.1103
		SSEVPTGSY	2.8708
		ASEDDIAAY	2.7231
		LSTDDGATY	2.0833
		STCTMDAAY	2.9323
		QLTLSSLFY	2.1468
		**LSSLFYLPY**	**3.3553**
		**NSSTDLLDY**	**3.0863**
		LLDYKAAQY	2.5937
		GSAGKSFNY	2.0306
**2**	**LdBPK_211410**	CTHASMALV	0.7588
		**YTEAQQLHT**	**1.4547**
		ATNWAQLAV	0.9146
		**WMDLHSNRL**	**1.0307**
		MTFLNTLLL	0.9509
		TVDEAVMSV	0.829
		AVDGSDATF	0.8317
**3**	**LdBPK_310120**	SAHVGSSVY	1.0079
		FMHPRMKTY	1.239
		**QVDWLVALY**	**3.1899**
		GQDGKVQGY	1.0879
		AAAVKSVEY	1.442
		DTEGKVRCY	1.7273
		LLNLNGMLY	2.1102
		VTTIKGGVY	2.4195
		YVFQTSTPY	1.2417
		TVGTRIRLY	1.0238
		**FTDSISLSF**	**3.2792**
		IIEPEAVQY	1.3645
**4**	**LdBPK_220130**	PAEDSNAIY	1.8882
		**KSETVSIQY**	**3.0954**
		DSATNQTYF	1.0139
		GSTGYDPAY	1.2708
		YINADHQRY	1.736
**5**	**LdBPK_310860**	SLQYANVTY	1.4446
		**SADFQHVPY**	**2.478**
		KVDMRDHFF	1.0015
		GAAGDAMLY	1.5074
		QTDARRSLQ	0.9877

Supplementary table 2: Predicted HTL epitopes for *Leishmania donovani* secretory proteins

S. No.	Accession no.	Alleles	Start	End	Sequence	Percentile
**1**	**LdBPK_190160**	H2-IAd	235	249	VESMQALQAASIVIA	0.03
		H2-IAb	221	235	FTSNVVYLPATATLV	0.2
		H2-IEd	761	775	QVDLASYVRGRRKWM	4.96
**2**	**LdBPK_211410**	H2-IAd	1	15	MCTHASMALVAAVAL	0.03
		H2-IAb	451	465	ATFSSWDASASSASN	1.04
		H2-IEd	127	141	LPASWGLLRNLRKVR	17.41
**3**	**LdBPK_310120**	H2-IAd	743	757	FNMHFMAAIKYTLIV	0.54
		H2-IAb	398	412	TNAAYALSTPLVINA	0.48
		H2-IEd	691	705	VGTRIRLYRERFHKP	9.64
**4**	**LdBPK_220130**	H2-IAd	210	224	LGVVKMIKAMKHSKG	0.55
		H2-IAb	72	86	FPSFHAGSKANVVIA	3.85
		H2-IEd	260	274	EVLHKKLSTSRSRRA	17.71
**5**	**LdBPK_310860**	H2-IAd	173	187	VALLAAVDVQAHVNR	0.19
		H2-IAb	247	261	HVPYEVYCAAAAGTN	1.8
		H2-IEd	135	149	FQRSYLAVRRTVRAA	12.78

Supplementary table 3: Predicted B cell epitopes for the final vaccine construct protein proteins

S. No.	Position	Epitope	Score
1	317	SSWDASASSASNGPGPGLPA	1
2	500	YCAAAAGTNGPGPGFQRSYL	1
3	386	INAGPGPGVGTRIRLYRERF	1
4	421	KAMKHSKGGPGPGFPSFHAG	1
5	456	LHKKLSTSRSRRAGPGPGVA	1
6	219	YSADFQHVPYGPGPGVESMQ	1
7	479	AVDVQAHVNRGPGPGHVPYE	1
8	262	PATATLVGPGPGQVDLASYV	1
9	241	QAASIVIAGPGPGFTSNVVY	1
10	360	AAIKYTLIVGPGPGTNAAYA	1
11	43	AAAGAAPAGAAVEAAEEQSE	0.999
12	339	GLLRNLRKVRGPGPGFNMHF	0.999
13	283	GRRKWMGPGPGMCTHASMAL	0.998

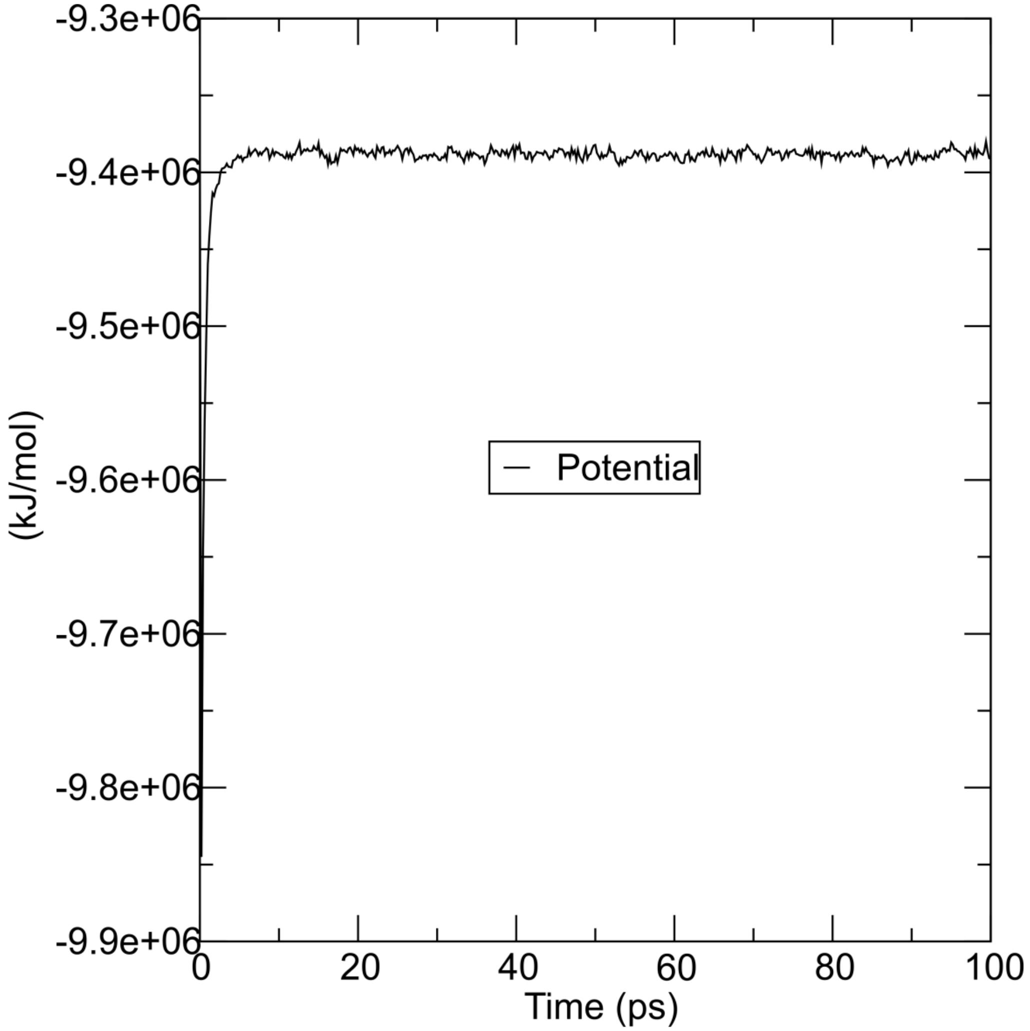

Supplementary figure1: Potential energy of protein ligand (TLR-4 vaccine) complex as a function of energy minimization step during molecular dynamics simulation.

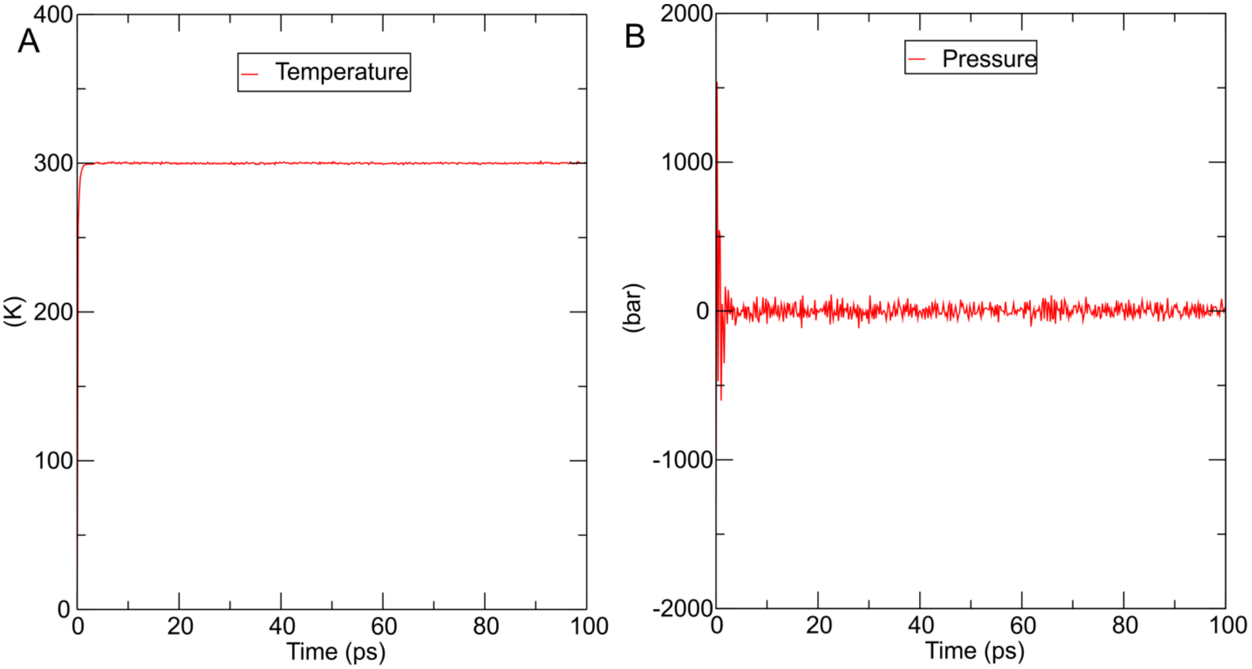

Supplementary figure 2: Molecular dynamics simulation study of protein ligand (TLR-4 vaccine) complex (A) Temperature progression curve indicating that temperature of the system reaches a up to 300K and remains stable for rest of the equilibration period (100 ps) (B) Pressure progression curve indicating pressure fluctuation up to 1bar.
